# Delayed Cerebral Ischemia of the Corpus Callosum: A Case Report

**DOI:** 10.7759/cureus.6379

**Published:** 2019-12-13

**Authors:** Dia R Halalmeh, Neil Klinger, Sherwin Azad, Hassan Fadel, Marc D Moisi

**Affiliations:** 1 Neurological Surgery, Detroit Medical Center, Detroit, USA; 2 Neurological Surgery, Wayne State University School of Medicine, Detroit, USA; 3 Surgery, University of California, San Francisco, USA; 4 Neurological Surgery, Henry Ford Health System, Detroit, USA

**Keywords:** corpus callosum, ventriculostomy, ischemia, intraventricular hemorrhage, intracerebral hemorrhage, delayed ischemia, infarct

## Abstract

Ischemic infarction of the corpus callosum is a rare condition due to its rich vascular supply and therefore has been infrequently reported. Here, we present a case of a patient who developed a delayed infarct of the corpus callosum in the body. The condition was characterized by bilateral lower extremity weakness and visual disturbances following intraventricular hemorrhage managed with ventriculostomy. Understanding the anatomy and function of the corpus callosum is crucial to understanding the etiology of infarctions as well as their clinical significance. It is also essential to distinguish between relatively common post-shunting changes and true infarction and to recognize the limited consequences of corpus callosum infarction. Increased awareness of this rare infarct would help to prevent unnecessary interventions and increase the ability of the physician to provide optimal care for the patient.

## Introduction

Delayed cerebral ischemia (DCI) is one of the most devastating complications of subarachnoid hemorrhage (SAH), often leading to morbidity and mortality. Occurring in up to 30% of patients, DCI is defined clinically as the presence of focal or global neurological deterioration, most commonly presenting between four and ten days following the initial ictus, and is supported with radiological evidence of a new infarct not present on admission [1]. The clinical features of DCI vary widely and may include the sudden presence of motor deficits such as hemiparesis, aphasia, or even a generalized decline in the level of the patient’s consciousness [1]. Although the precise mechanisms of DCI are unknown, it is thought to be mediated via circulating hemoglobin by-products, with recent studies showing the amount of ventricular blood or presence of intracerebral hemorrhage (ICH) as independent risk factors for DCI infarction [2,3].

Conversely, primary ICH most commonly affects the structures of the basal ganglia and represents a devastating consequence of uncontrolled malignant hypertension among other pathologies including vascular malformations, cerebral amyloid angiopathy, and trauma. Although distinct from SAH in both etiology and pathology, ICH may evolve and extend into the ventricular system, portending poor outcomes [4]. Given that the occurrence of DCI has been linked with the amount of ventricular and subarachnoid blood as well as its by-products, patients suffering from primary ICH with substantial secondary IVH may experience complications akin to those seen in SAH patients, and maybe similarly predisposed to the development of DCI. However, the association between DCI and isolated ICH with IVH has yet to be thoroughly investigated.

There is no characteristic radiological or clinical presentation of DCI, although a recent study found that infarctions due to DCI most commonly occur in the deep branches of the anterior, middle, and posterior cerebral arteries supplying the caudate, putamen, thalamus, and corpus callosum [5]. The predilection of DCI infarctions to affect vessels supplying the corpus callosum, in particular, is unique given that the corpus callosum receives a rich vascular supply from both the anterior and posterior cerebral circulation. As such, ischemia of the corpus callosum is a rare condition that has been infrequently reported [6-11]. Here, we present the case of a 29-year-old male who suffered a hypertensive ICH with significant extension into the ventricular system and subsequently developed DCI with an infarction affecting the body of the corpus callosum several days later. To the best of our knowledge, this is the first-ever reported case of an isolated corpus callosum infarction due to DCI.

## Case presentation

**Figure 1:**
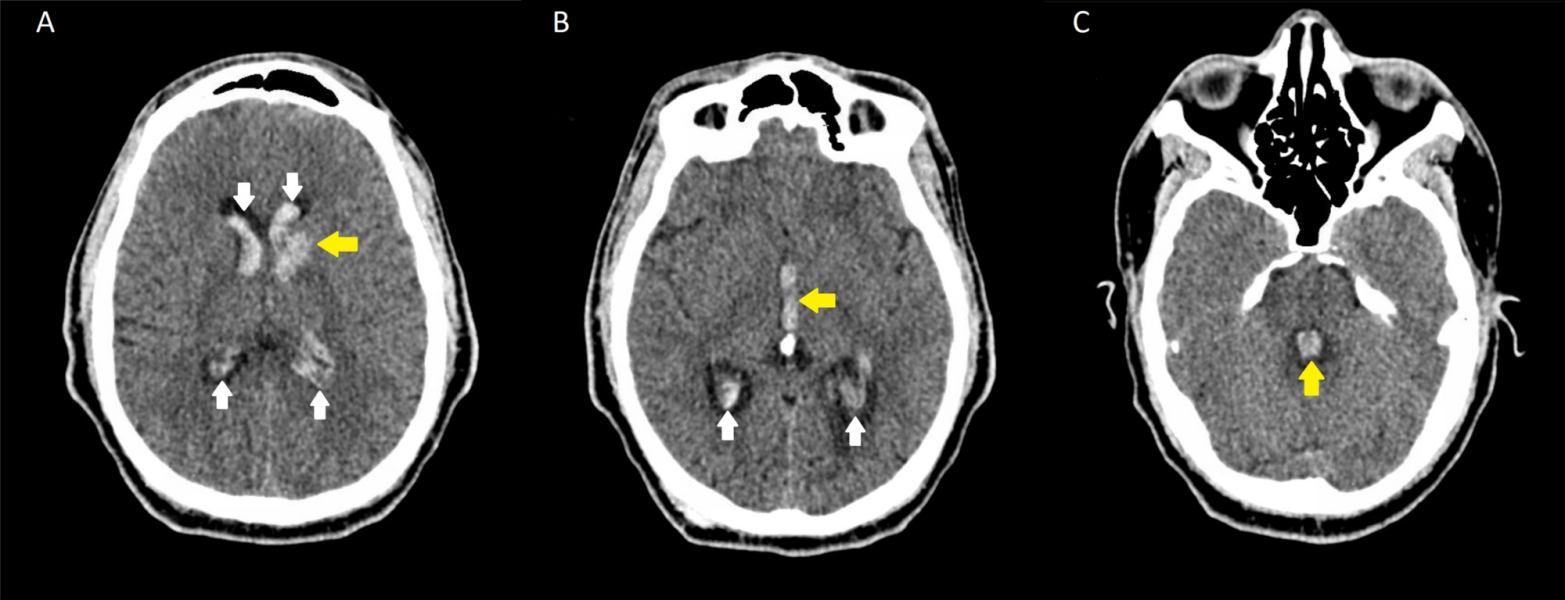
Intraparenchymal hemorrhage with intraventricular extension CT of the head without contrast demonstrating (A) intraparenchymal hemorrhage in the left caudate head (yellow arrow in A) with intraventricular extension into both lateral ventricles (white arrows in A and B) as well as (B) the third (yellow arrow) and (C) fourth ventricles (yellow arrow)

Our patient is a 29-year-old male with a past medical history significant for type 2 diabetes, untreated hypertension, chronic kidney disease, and peripheral arterial occlusive disease. He presented to the emergency department with an acutely worsening occipital headache. He had been experiencing a headache for several days prior to admission, which had abruptly worsened. He also endorsed feeling increasingly lethargic and was suffering from severe stomach pain associated with several episodes of non-bloody non-bilious emesis. At presentation, he appeared to be in acute distress, was tachycardic, tachypneic, and hypertensive with a blood pressure of 282/136 mmHg. No focal neurologic deficits were appreciated on examination. Shortly following admission, he developed altered mental status and increasingly labored respirations requiring urgent intubation. Initial CT of the brain demonstrated an acute hemorrhage in the left caudate consistent with hypertensive intraparenchymal hemorrhage (IPH) with extension into the lateral ventricles bilaterally, as well as the third and fourth ventricles (Figure 1). CTA of the head and neck was negative for vascular pathologies. 

**Figure 2:**
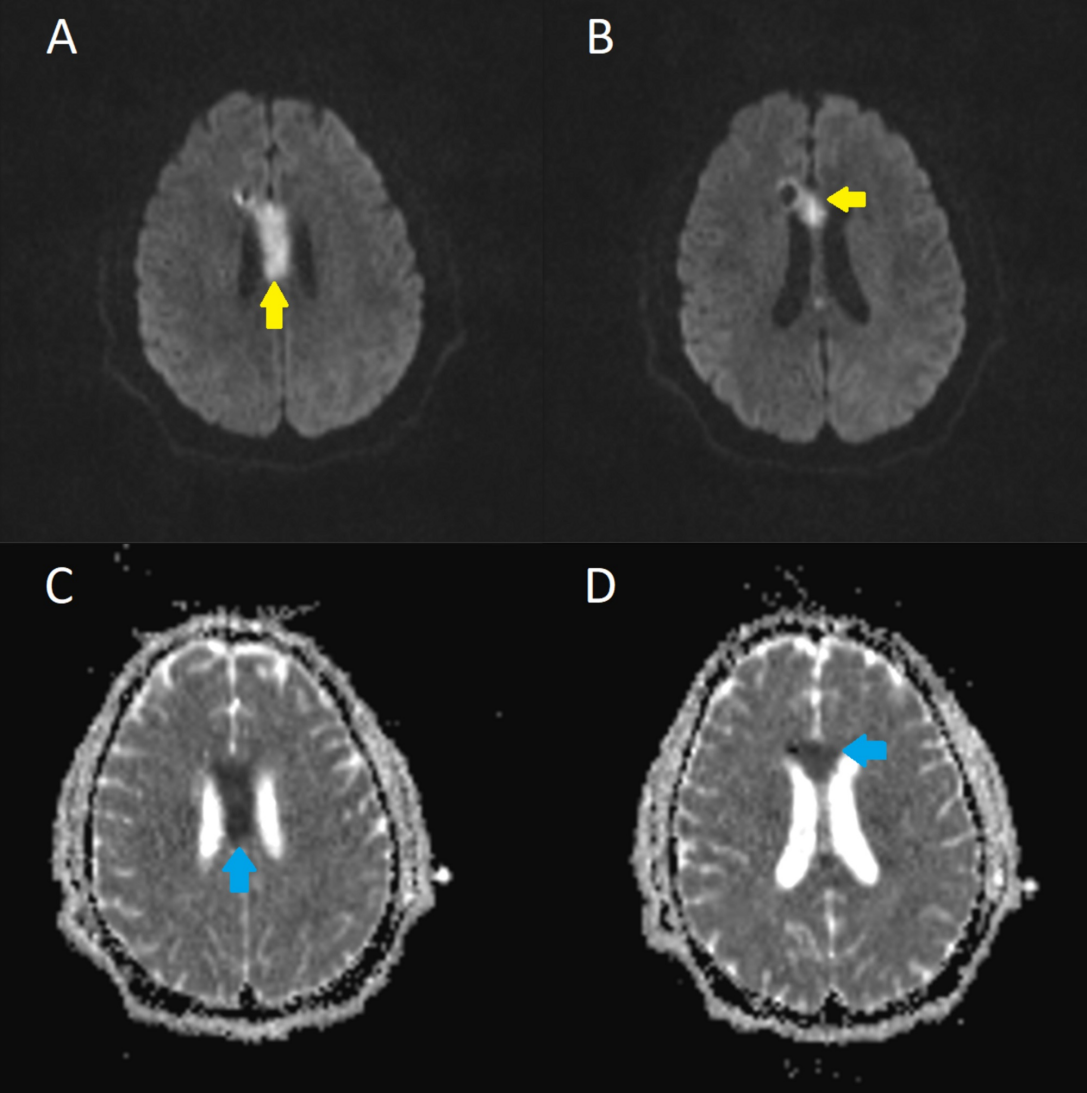
Infarction of the corpus callosum MRI showing areas of hyperintensity in the body (A) and genu (B) of the corpus callosum (yellow arrows) on DWI with restricted diffusion (blue arrows) on respective ADC mapping (C and D), representing a recent ischemic insult DWI: diffusion-weighted imaging; ADC: absolute diffusion coefficient

An external ventricular drain (EVD) was emergently placed in a single pass using the right frontal approach without complication, with the ventriculostomy catheter visualized terminating at the foramen of Monro on postoperative imaging. Three days following EVD placement, a one-time injection of 1 mg of the tissue plasminogen activator (tPA) alteplase was administered intraventricularly to aid in clot resolution. His recovery continued without complication until post-procedure day seven when he complained of increasing lethargy and experienced altered mentation. This was followed by an acute onset of significant bilateral lower extremity paresis (2/5 hip flexion strength bilaterally, down from full strength at baseline). Emergent non-contrast CT imaging did not reveal any significant changes, including any evidence of new hemorrhage or ischemia. A follow-up MRI revealed an acute ischemic infarction of the body of the corpus callosum (Figure 2). Requiring aggressive inpatient rehabilitation, the patient’s hospital course was further complicated by acute on chronic kidney injury requiring dialysis, resistant hypertension, urinary retention, and hematemesis. Upon discharge, the patient recovered baseline mentation but had persistent yet greatly improved bilateral lower extremity weakness (4/5 hip flexion strength bilaterally) for which he was released to a rehabilitation facility. 

## Discussion

DCI is recognized as one of the most feared causes of morbidity and mortality among patients surviving subarachnoid hemorrhage. Historically attributed to cerebral vasospasm, the etiology of DCI is now thought to result from multifactorial pathophysiology consisting of vasospasm as well as neuro-inflammation, microvascular vasospasm, microthrombosis, cerebral dysregulation, and cortical spreading depression [12]. Patients suffering from IVH, although distinct from SAH, have been shown to carry a similar predisposition to DCI as SAH patients, with several studies reporting the incidence of secondary cerebral infarctions following either primary or secondary IVH [2,12,13,14]. The presence of DCI in both SAH and IVH patients is hypothesized to be because both SAH and IVH patients have blood and blood degradation products distributing to the subarachnoid space, with the severity of IVH correlated with an increased risk of DCI [2,15]. This hypothesis is supported in the literature, particularly in a study by Claassen et al. (2001) that investigated the risk factors and predispositions of DCI development in 276 SAH patients with and without IVH and/or ICH. The study’s multivariate analysis found that IVH, especially when involving the ventricles bilaterally, was the strongest independent predictor for the development of DCI infarction [2]. The association between the presence and amount of ventricular blood and DCI has been subsequently expanded upon by studies finding ICH to be also associated with DCI in SAH patients [3]. Our patient, suffering from both ICH and substantial bilateral IVH, was therefore significantly predisposed to the development of DCI, clinically manifesting as altered mental status and bilateral lower extremity paresis following seven days of otherwise uncomplicated recovery.

Our patient suffered an infarction to the body of the corpus callosum, which derives its main blood supply via the median callosal artery, a perforating artery of the pericallosal artery, itself an extension of the anterior cerebral artery (ACA) [7]. Although no such DCI-related infarction has been reported in the literature, recent studies concerning the location of cerebral infarctions secondary to DCI help explain the etiology of our patient’s unique case. Of the 126 SAH patients analyzed in a prospective study by Wong et al. (2015), the majority of DCI infarctions occurred in the deep penetrating branches of the anterior, middle, and posterior cerebral arteries, which included the pericallosal artery and its associated perforating arteries that directly supply the corpus callosum without collaterals [5,7]. On subsequent univariate analyses, such DCI infarctions of the penetrating arteries were also associated with poorer modified Rankin Scale, Montreal Cognitive Assessment, and Mini-Mental Status Examination results and thus a poorer prognosis [5]. The tendency of DCI infarctions to occur in deep penetrating arteries is supported by Rabinstein et al. (2004), who, in studying the topographical pattern of DCI infarctions in SAH patients, found that isolated deep infarcts, although not the most common among the study population, occurred in 18% of patients, with 60% of such lesions being symptomatic [16]. Rabinstein et al. also found that patients with single infarctions frequently had ischemia in the territory of the ruptured aneurysm (79%), thereby establishing a relationship between the location of the infarct and the location of the hemorrhage. The findings of these studies demonstrate that DCI infarctions are more likely to occur in deep penetrating arteries, have been shown to occur as isolated deep infarctions, and maybe regionally associated with a local site of hemorrhage, all of which is consistent with our patient who we believe developed DCI affecting a perforating artery of the pericallosal artery with concurrent proximate ICH formation and bilateral IVH, leading to an isolated infarction of the body of the corpus callosum. These findings are augmented by a prospective report by Alba et al. that investigated the relationship between SAH aneurysm location and the incidence of vasospasm in 250 patients [17]. The study found that, although radiographic and/or clinical vasospasm occurred in 50.7% of total participants, patients with pericallosal aneurysms had the highest rate of vasospasm (77%) [17]. Furthermore, symptomatic vasospasm (defined by the authors as DCI leading to clinical deterioration) occurred most often in patients with pericallosal aneurysms (56%) as compared to an incidence of 22% in all other locations, potentially due to the smaller diameter of the pericallosal arteries at baseline [17]. The pericallosal artery and its distal branches, which we believe were affected in our patient, are particularly predisposed to the development of DCI. Thus, we infer that our patient with IVH and ICH may have suffered delayed infarction to the body of the corpus callosum as a consequence of pericallosal artery involvement.

The predilection of DCI infarctions to affect vessels supplying the corpus callosum, in particular, is unique given that ischemia of the corpus callosum is an incredibly rare condition that has been infrequently reported [6-11]. The paucity of such infarctions is due to the rich vascular supply of the corpus callosum, which is derived from both the anterior and posterior cerebral circulation from each hemisphere. Further complicating our case is the lack of a consistent clinical presentation seen in patients with corpus callosum infarctions. Because the corpus callosum integrally contributes to the motor, executive, and cognitive pathways among others, the clinical syndromes that are encountered following corpus callosum infarction vary widely depending on the affected region as well as the degree to which the connectivity of neighboring structures are affected [18]. Reports have described corpus callosum lesions leading to a variety of clinical symptoms including amnesia [19], aphasias, and gait disturbances or weakness in the lower limbs [20], the last of which was a predominant symptom of our patient, who suffered an acute bilateral lower extremity paresis coupled with an altered mental status. Although the sudden presence of focal motor deficits together with a more generalized decline in the level of patient consciousness has been shown to be relatively characteristic of DCI infarctions, our patient’s unique presentation has never been reported before.

## Conclusions

Although rare, our patient’s case of a DCI infarction isolated to the corpus callosum seven days following spontaneous ICH with severe IVH serves as an example of DCI affecting non-SAH patients. With DCI being responsible for a majority of morbidity and mortality in patients suffering from SAH and, potentially, IVH, our report would suggest that urgent systematic screening is warranted in any patient with signs or symptoms of DCI occurring within two weeks of either primary or secondary IVH.
